# Machine Learning Model for Identifying Antioxidant Proteins Using Features Calculated from Primary Sequences

**DOI:** 10.3390/biology9100325

**Published:** 2020-10-06

**Authors:** Luu Ho Thanh Lam, Ngoc Hoang Le, Le Van Tuan, Ho Tran Ban, Truong Nguyen Khanh Hung, Ngan Thi Kim Nguyen, Luong Huu Dang, Nguyen Quoc Khanh Le

**Affiliations:** 1 International Master/PhD Program in Medicine, College of Medicine, Taipei Medical University, Taipei City 110, Taiwan; luuhothanhlam2013@gmail.com (L.H.T.L.); drhung.bvcr@gmail.com (T.N.K.H.); 2 Children’s Hospital 2, Ho Chi Minh City 70000, Vietnam; 3 Graduate Institute of Biomedical Materials and Tissue Engineering, College of Biomedical Engineering, Taipei Medical University, Taipei City 110, Taiwan; lengochoang252@gmail.com; 4 Orthopedic and Trauma Department, Cho Ray Hospital, Ho Chi Minh City 70000, Vietnam; drlevantuan.cr@gmail.com; 5 Department of Pediatric Surgery, University of Medicine and Pharmacy, Ho Chi Minh City 70000, Vietnam; hotranban@ump.edu.vn; 6 School of Nutrition and Health Sciences, Taipei Medical University, Taipei City 110, Taiwan; kimngan1702@gmail.com; 7 Department of Otolaryngology, University of Medicine and Pharmacy, Ho Chi Minh City 70000, Vietnam; luonghuudang167@gmail.com; 8 Professional Master Program in Artificial Intelligence in Medicine, College of Medicine, Taipei Medical University, Taipei City 106, Taiwan; 9 Research Center for Artificial Intelligence in Medicine, Taipei Medical University, Taipei City 106, Taiwan

**Keywords:** antioxidant proteins, machine learning, Random Forest, protein sequencing, feature selection, computational modeling

## Abstract

**Simple Summary:**

Antioxidant compounds protect the human body from many kinds of diseases as well as the degeneration of age. Several micronutrients that were found in the last century such as vitamins A, C, and E have become popular in our life. Scientists are trying to find more and more antioxidant compounds not only from experimenting in the laboratory but also from assisting by the computer. Our research utilized a computational method for the swift and economic identification of antioxidant compounds. The research presents a predictor that got a high accuracy of 84.6% for the detection of antioxidants. Therefore, our predictor is promising to be a useful tool to discover a new antioxidant compound.

**Abstract:**

Antioxidant proteins are involved importantly in many aspects of cellular life activities. They protect the cell and DNA from oxidative substances (such as peroxide, nitric oxide, oxygen-free radicals, etc.) which are known as reactive oxygen species (ROS). Free radical generation and antioxidant defenses are opposing factors in the human body and the balance between them is necessary to maintain a healthy body. An unhealthy routine or the degeneration of age can break the balance, leading to more ROS than antioxidants, causing damage to health. In general, the antioxidant mechanism is the combination of antioxidant molecules and ROS in a one-electron reaction. Creating computational models to promptly identify antioxidant candidates is essential in supporting antioxidant detection experiments in the laboratory. In this study, we proposed a machine learning-based model for this prediction purpose from a benchmark set of sequencing data. The experiments were conducted by using 10-fold cross-validation on the training process and validated by three different independent datasets. Different machine learning and deep learning algorithms have been evaluated on an optimal set of sequence features. Among them, Random Forest has been identified as the best model to identify antioxidant proteins with the highest performance. Our optimal model achieved high accuracy of 84.6%, as well as a balance in sensitivity (81.5%) and specificity (85.1%) for antioxidant protein identification on the training dataset. The performance results from different independent datasets also showed the significance in our model compared to previously published works on antioxidant protein identification.

## 1. Introduction

Oxygen is an indispensable element in normal life which is hailed as the “Elixir of Life”—a great tonic for medical therapies [1]. However, in certain cases, it is reported that oxygen is also one of the factors that adversely affect human health due to the formation of reactive oxygen species (ROS) and free radicals [2]. Free radicals or ROS are derived from essential metabolic processes in the internal human body and play an important role in cellular communication and homeostasis [3]. The attendance of an unpaired electron in free-radicals is normally unstable and highly reactive, leading to the aggression between them and important macromolecules (i.e., lipids, nucleic acids, and proteins) in the intracellular environment. In general, the mechanism of antioxidant activity starts when the antioxidant compounds react in one-electron reaction with free radicals, and then prevent oxidative damage. The antioxidant molecules react to free radicals in two ways: the small-molecule antioxidants neutralize the free radicals in a process called radical scavenging and carry them away, or the large-molecule antioxidants absorb free radicals and prevent them from destroying important macromolecules. The small-molecule antioxidants are micronutrients such as vitamin C, vitamin E, carotenoids, and glutathione (GSH), while the large-molecule antioxidants are enzymes superoxide dismutase (SOD), catalase (CAT), glutathione peroxidase (GSH-PX), and proteins (albumin) [4].

The concentration of free radicals can rapidly increase due to external factors such as X-rays, cigarette smoke, industrial chemicals, food preservatives, or environmental pollution [5]. The extension of free radical concentration in the human body can create an imbalance between antioxidants and free radicals (this phenomenon is called oxidative stress) [2]. It is worth mentioning that the oxidative stress also plays an important role in accelerating the aging process, metabolic syndromes [6] as well as the cause of many dangerous human diseases such as sclerosis [7], various inflammation-reactions (arthritis, vasculitis, glomerulonephritis, lupus erythematous, adult respiratory diseases syndrome) [8,9], cardiovascular diseases (heart diseases, stroke) [10], and cancer [9,11].

In the early literature, the term antioxidant in biology originally was used to refer specifically to the prevention of the oxidation of unsaturated fats [12]. Over the decades, the term has been used increasingly in research reports on the self-production of antioxidants in the human body [2,3,13]. Besides categorizing into molecular sizes, antioxidants can be also classified based on its activity: enzymatic and non-enzymatic antioxidants. Enzymatic antioxidants are SOD, CAT, GSH-PX as mentioned above, while non-enzymatic antioxidants are uric acid, bilirubin, metallothionein, micronutrients, or plant polyphenols [4]. However, due to the current living-environment or daily habits [7], the proportion of free radicals in the human body is often greater than the number of antioxidants naturally produced. Hence, the identification of many exogenous micronutrients with antioxidant properties such as Vitamin A, C, and E [2] revolutionized a huge amount of studies about antioxidants and led to the applications of them in medical-treatment therapies.

Although a wide range of different biochemical tests exists, as well as rapid aid-kits that can be used to determine the properties of antioxidant proteins, the process is very time-consuming [14] and high cost. Therefore, the development of computational models based on computer tools to assist in the accurate prediction of antioxidant proteins is highly desirable. In 2013, Feng et al. [15] had carried out the Naïve Bayes classifier by using the features selection method to detect antioxidant proteins with an accuracy of 55.85%. Then, to save computational time, they reduced the feature dimension to 44, and the maximum accuracy achieved was increased from about 55% to 66.89% with 10-fold cross-validation training processes. Three years later, Feng et al. [16] published another research using the same dataset. These authors developed an antioxidant predictor, namely AodPred, by using the support vector machine (SVM) method with optimal 3-gap dipeptides composition. As shown in their studies, AodPred improved the prediction accuracy to 74.79% after the thorough jackknife cross-validation tests. Compared to the accuracy results of previous researches, Xu et al. [17] had presented a computational method called “SeqSVM” in 2018 to predict antioxidant proteins, with the primary sequence features that were observed mainly from the physicochemical properties and sequence information of the protein. The dimensions of the extracted features were 188 with an accuracy of 89.46%. Recently, Li et al. [18] presented a special prediction method with the number of prediction models up to 15 support vector machine (SVM) models. They excluded six models with low sensitivity (<20%), and then the validation results showed from remained 9/15 models were optimally built with accuracy were all greater than 87%. Their best model, called Vote9, achieved a high accuracy of 94.2%, sensitivity of 65%, and specificity of 99%.

In addition, the enormous amount of antioxidant data, as well as the wide range of common databases for protein properties [5], have prompted us to develop a new machine learning model based on a variety of sequencing properties of proteins to produce an aid-tool that has a higher rate of accurate prediction. In this study, we established a prediction model based on the same benchmarking (training) dataset with Butt et al. [5] to compare the efficiency of applying more features to the prediction model including 249 positive and 1531 negative antioxidant protein sequences. With the benchmarking dataset, we obtained nine feature sets with an accuracy higher than 70% throughout screening 17 feature sets by the Random Forest classifier. Those obtained feature sets were combined according to three different methods to achieve new hybrid feature sets (F1, F2, and F3). Finally, we processed those hybrid feature sets with different algorithms including Random Forest (RF), Sequential Minimal Optimization (SMO), J48, and Deep Learning (DL) to find the best model.

## 2. Materials and Methods

**Figure 1:**
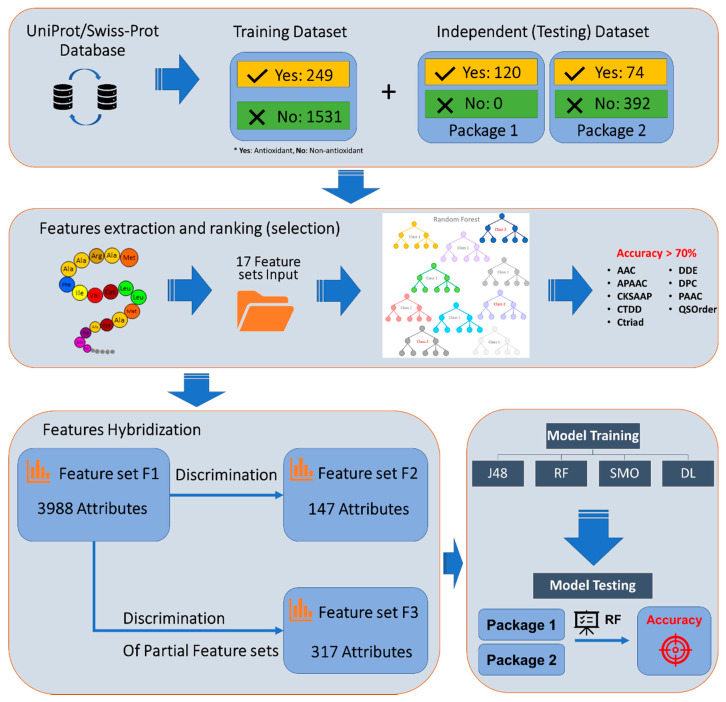
The flowchart summarizes the research processes.

We summarize our research progress in Figure 1. There are four sub-processes in our whole architecture including data collection, feature extraction, feature selection, and model construction. All of them are described one-by-one in the following sections.

### 2.1. Dataset Preparation

In 2019, a recent article has been published to predict the antioxidant proteins using statistical moment-based features [5]. In this aforementioned study, the dataset was gathered from the UniProtKB/Swiss-Prot database (version released in 2018_05, http://www.uniprot.org) and to the best of our knowledge, it is the latest benchmark dataset for antioxidant protein prediction. To facilitate the comparison between our model and the previous work, we used the same benchmarking (training) dataset that was collected from their research. The collected dataset contains overall 1900 protein sequences, including 369 positive (antioxidant) and 1531 negative (non-antioxidant) sequence samples.

In the following step, the positive dataset was also separated into two parts with the approximate ratio of 2:1 (249 samples as a training dataset and 120 samples as an independent test set). Moreover, we also used 1531 non-antioxidant protein sequences as a negative dataset to incorporate with the 249 positive samples in the training process. Subsequently, the positive data set is denoted as “yes”, and vice versa, the negative data was labeled as “no”.

To enhance the prediction model in our research, and also compare with previous studies, we exploited 2 different validation datasets: (1) the package 1 was prepared as same as the dataset constructed in [5] with only 120 positive samples; the package 2 included 74 positive samples and 392 negative samples following Zhang et al. [19]. Finally, we also manually collected a novel set of antioxidant proteins from the UniProt (TrEMBL) database to serve as an external set for validating the potential of our models on newly discovered proteins. Specifically, we used the keyword searching “antioxidant”, and then we chose only 2934 sequences that were discovered from January to August 2020. By this criterion, we can gather antioxidant protein sequences that did not appear in any of our training data as well as the other two independent datasets. As mentioned in the UniProt document, most keywords were added in the manual annotation process using different sources, thus retrieving data using “keywords” function was reliable. Therefore, we were highly confident that our validation set contained newly discovered antioxidant proteins.

### 2.2. Feature Extraction

iLearn [20] and iFeature [21] are open-source, effective, and convenience toolkits, which was performed based on the Python environment (Python version 3.0 or above). They are commonly used to generate descriptors for various molecular structures such as DNA, RNA, or protein/peptide sequences. In this research, we used both iLearn and iFeature to extract protein sequences features descriptor based on seven following groups:

- ***Amino acid composition***: The amino acid composition (ACC) is defined as the number of amino acids of each type normalized with the total number of residues [22]. With this descriptor group, we not only encoded the frequency of each amino acid in the peptide/protein sequences by using the ACC descriptor but also combined with the composition of k-spaced amino acid pairs (CKSAAP), dipeptide composition (DPC), dipeptide deviation from the expected mean (DDE), subsequently.
- ***Composition/Transition/Distribution (C/T/D)***: this descriptor group represents the distribution pattern of the amino acid-based on the specific structural or physicochemical property of that peptide/protein sequence [23,24]. Seven types of physical properties have been used for calculating these features: hydrophobicity, normalized ***Van der Waals*** volume, polarity, polarizability, charge, secondary structures, and solvent accessibility.
- ***Conjoint triad***: This feature extractor can be used for exploring the properties of each amino acid in protein sequences and its vicinal amino acids by relating any three continuous amino acids as a unique unit [25].
- ***Autocorrelation:*** The autocorrelation descriptor was firstly introduced by Moreau and Broto, which is mainly based on the distribution of amino acid properties along the sequence [26]. In this group, there are three autocorrelation descriptors including Geary, Moran, and Normalized Moreau-Broto (NMBroto).
- ***Pseudo-amino acid composition***: The pseudo amino acid composition (PseAAC) was originally introduced by Chou in 2001 [27] to represent protein samples for improving the prediction of membrane proteins and protein subcellular localization. Compare to the original AAC method, it also characterizes the protein mainly using a matrix of amino-acid frequencies of the peptide/protein sequences without significant sequential homology to other proteins.
- ***Grouped amino acid composition*:** With this descriptor group, the features are extracted with the encoding and categorizing into five classes based on their physicochemical properties such as hydrophobicity, charge, or molecular size [21].
- ***Quasi-sequence-order***: This promising descriptor can pass through the extreme complication of the peptide/protein sequences (permutations and combinations) based on the augmented covariant discriminant algorithm. It also allows us to approach the higher prediction quality with various protein features as well [28].

Here, over 4000 dimensions (attributes) in total have been extracted by using the seven descriptors described in Table 1.

### 2.3. Feature Selection and Attribute Discrimination

#### 2.3.1. Feature Selection

**Figure 2:**
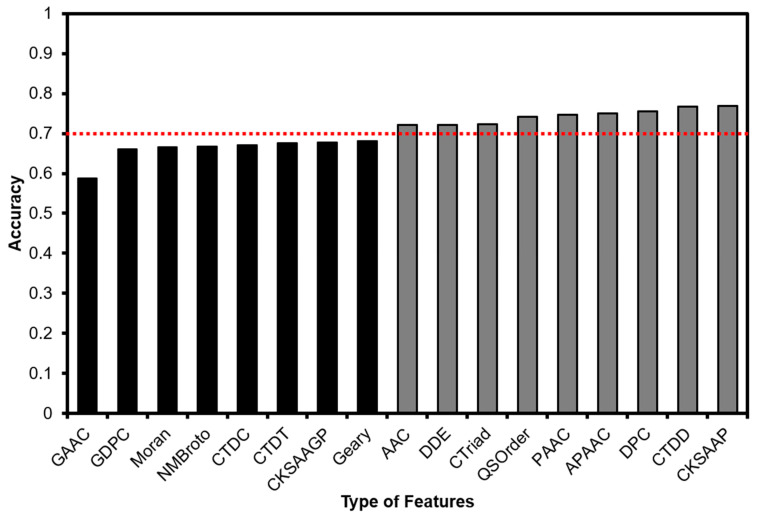
Feature selection processing with the Random Forest algorithm. The feature sets in gray color show an accuracy higher than 70%.

The first important step is to identify the optimal features that contribute to correctly classifying antioxidant and non-antioxidant protein sequences. Meanwhile, we utilized the Random Forest algorithm to identify which feature sets gave the highest accuracy when predicting the antioxidant activity of protein sequences. Cost-Sensitive matrices have been applied to adjust the complication of imbalance in our training dataset. This step has been performed with the implementation of the Waikato Environment for Knowledge Analysis (WEKA) library [29]. Then, we selected nine feature sets including AAC, DDE, CTriad, CTDD, CKSAAP, DPC, QSOrder, PAAC, and APAAC (Figure 2) to use in the following steps.

#### 2.3.2. Attribute Discrimination

The number of attributes in each of the nine feature sets was reduced by sequentially applying the WEKA Attribute Subset Evaluator and the BestFirst Searching Method tools:

- Attribute Evaluator—Correlation-based feature subset selection (CfsSubsetEval): This evaluator was firstly introduced in 1998 by Hall et al. [30], that can assume the value of the subset attributes by considering the individual predictive ability of each feature compared with the degree of redundancy between them.
- Searching method—BestFirst”: This tool can examine the space of attribute subsets by greedy hill climbing augmented with a backtracking facility. An important notice here is that “BestFirst” may be searching from both directions: starts with the empty set of attributes and search forward, or begins the opposite with the full set of attributes or any random point.

Finally, we obtained nine feature sets, each with a reduced number of attributes.

### 2.4. Model Training

First, to comprehensively investigate all features in model construction as well as evaluate the efficiency of the attribute discrimination, we tried building our model in different feature datasets as follow:

- Feature set F1: Hybridization of all nine original feature sets together. This feature set contains 3988 attributes.
- Feature set F2: We applied the “select attributes” function of Weka (developed by the University of Waikato, Hamilton, New Zealand, USA) software on Feature F1 data. This hybrid feature set contained 147 attributes.
- Feature set F3: Attributes were reduced by filtering each of the nine feature sets separately and then combining them to create a hybridized set of 317 attributes.

With three collected hybrid features (F1, F2, F3), we applied both machine learning and deep learning methods to develop the comprehensive prediction model with high accuracy. In this study, we used three different algorithms in the machine learning method including Random Forest, J48, and SMO, while we chose the “Deeplearning4j” programming library for the Deep Learning method.

#### 2.4.1. Random Forrest (RF)

RF is an ensemble learning algorithm in the Weka software that was introduced by Leo Breiman [31]. This statistical learning methodology was used for classification, regression, and other tasks that operate by generating a multi of random trees at training time and outputting the class that is the mode of the classes (classification) or mean prediction (regression) of the individual trees in the forest. The optimal parameters for RF in Weka have been set as follows: num_lteration (the number of trees in the random forest) equals to 500; numFeatures (sets the number of randomly chosen attributes) is calculated by the square root of the number of attributes in the dataset (i.e., Feature set F1 contains 3988 attributes, so the number of features are $\sqrt{3988}$ = 63).

#### 2.4.2. J48

J48 (also known by the name C4.5) is an algorithm used to generate a decision tree developed by John Quinlan [32]. The decision trees generated by J48 are commonly used for classification as a statistical classifier. In 2011, the author of Weka machine learning software described the J48 (C4.5) algorithm as “a landmark decision tree program that is probably the machine learning workhorse most widely used in practice to date”. In J48, we set the confidenceFactor (the confidence factor used for pruning) to 0.25 and the minNumObj (the minimum number of instances per leaf) to 2.0.

#### 2.4.3. Sequential Minimal Optimization (SMO)

Sequential minimal optimization (SMO) is an algorithm developed by John Platt in 1998 at Microsoft Research to solve the quadratic programming (QP) problem that arises during the training of support vector machines (SVM) [33]. The optimization process was often executed by breaking the problem into a series of smallest possible sub-problems, which are then solved analytically. For SMO, C (the complexity parameter C) was set to 1.0; epsilon was set to 1.0×E−12; calibrator (the calibration method) was Logistic -R 1.0E−8 -M -1 -num-decimal-places 4.

#### 2.4.4. Deep Learning (DL)

Deeplearing4j or Eclipse Deeplearning4j is the deep learning programming library written by Java and it was also integrated into the Weka program [34]. Deeplearning4j framework includes a series of implemented algorithms such as the restricted Boltzmann machine, deep belief net, deep autoencoder, stacked denoising autoencoder, and recursive neural tensor network, word2vec, doc2vec, and GloVe. For the specifications and layers in deep learning, we used a fully connected layer with an output layer (softmax activation function and MCXENT loss function), the number of GPUs was set at one, and the size of the prefetch buffer for multiple GPUs was set at 24.

### 2.5. Model Performance Evaluation

All of the aforementioned algorithms were executed by using Weka with 10-fold cross-validation. To evaluate the performance of all constructed models in both machine learning and deep learning methods, we used five measurements that were commonly used in binary classification tasks [35,36] (all data was encoded) including sensitivity, specificity, accuracy, Matthews correlation coefficient (*MCC*) and Area Under the Receiver Operating Curve (AUC). These evaluation measurements are defined as follows:

(1) $$Sensitivity=\frac{TP}{TP+FN}$$

(2) $$Specificity=\frac{TN}{TN+FP}$$

(3) $$Accuracy=\frac{TP+TN}{TP+FP+TN+FN}$$

(4) $$MCC=\frac{TP*TN-FP*FN}{\sqrt{\left(TP+FP\right)\left(TP+FN\right)\left(TN+FP\right)\left(TN+FN\right)}}$$

where *TP*, *TN*, *FP*, and *FN* respectively denote true positives, true negatives, false positives, and false negatives.

Moreover, to show the efficiency of the applied model in this study, we compared our antioxidant proteins predictor with the results of two similar studies from Butt et al. [5] and Zhang et al. [19]. These studies are similar topics with us in building an antioxidant protein predictor. In the testing process of models, we used the same independent dataset with these authors, including the Independent dataset 1 of Butt et al. [5], and Independent dataset 2 of Zhang et al. [19]. In spite of imbalance data on both antioxidant and non-antioxidant sequences, we also did not adjust data disparity. For equal comparison, we run testing datasets on our model without adjusting imbalance data. Then, we also double-checked again the testing datasets on our model with adjusting imbalance as a reference data.

## 3. Results

### 3.1. Features Selection

Our benchmarking dataset is an imbalance between positive samples (249 sequences) and negative samples (1531 sequences); it made us use “The Cost-Sensitive Classifier” function in WEKA software to get a balance in sensitivity and specificity result. In the first step, we had to determine the optimal cost-sensitive matrix in our model by evaluating different matrices. Finally, the cost-sensitive matrix of (1:35, 2:1) was superior to solve the imbalance data, so we determined it as our optimal matrix. Next, we put all 17 extracted feature sets into the Random Forest algorithm. We then excluded feature sets that have accuracy less than 70%, this choosing left nine feature sets: AAC, APAAC, CKSAAP, CTDD, CTriad, DDE, DPC, PAAC, and QSOrder as in Figure 2.

### 3.2. Model Performance Among Different Features and Algorithms

Before applying algorithms on the feature sets, we utilized matrices to calibrate imbalanced data in the Cost-Sensitive Classifier function of the Weka program. We tried to execute many matrices for each algorithm to achieve a balance between sensitivity and specificity. These matrices manipulated feature datasets F1, F2, F3. The most suitable matrix for each algorithm is described below:

- Feature dataset F1: (1:35; 2:1) for SMO and RF; (1:50; 2:1) for J48; (1:4; 2:1) for DL. However, we were unable to strike the balance when applying Feature set F1 on the SMO algorithm even though we tried many matrices: (1:25; 2:1), (1:30; 2:1), (1:35; 2:1), (1:35; 3:1), (1:35; 4:1), (1:40; 2:1). These matrices presented similar results so we chose the matrix (1:35; 2:1) for representation.
- Feature dataset F2: (1:10; 2:1) for SMO; (1:35; 2:1) for J48, RF, and DL.
- Feature dataset F3: the same matrix (1:35; 2:1) for all algorithms SMO, J48, RF, DL.

**Figure 3:**
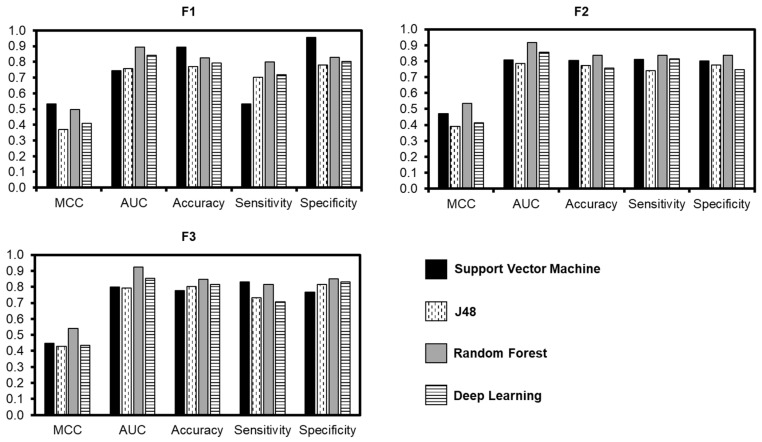
Evaluation of candidate models achieved from applying feature F1, F2, F3 on algorithms: SMO, J48, RF, and DL.

Next, all models were built on three different feature datasets (F1, F2, and F3). The result of prediction models in both machine learning and deep learning are showed in Figure 3 based on five measurements of binary classification.

Compare with the other feature datasets, feature F1 showed the highest efficiency when using SMO with the accuracy reach to 89.5%, while RF, DL, and J48 models were 82.5%, 79.2%, and 77%, respectively. However, the sensitivity and specificity from SMO showed the disparity values (53.4% and 95.4%), compared to the equivalent result in the RF model (79.9% and 82.9%)

In addition, the feature F3 showed the advantages when applied with models RF, J48, and DL to achieve high accuracy than using F1 and F2. The accuracy of RF was 84.6% along with DL was 81.5%, and J48 was 80.2%. Importantly, all these algorithms were also equivalent on the sensitivity (RF 81.5%; DL 70.7%; J48 73.1%; SMO 83.1%) and the specificity (RF 85.1%; DL 83.2%; J48 81.4%; SMO 76.8%). These results indicated that the Random Forest algorithm is the optimal model in our research with the feature set F3 used as a benchmarking dataset.

### 3.3. Comparision with Previous Methodologies

#### 3.3.1. Comparison with Existing Models in the Cross-Validation Test

**Figure 4:**
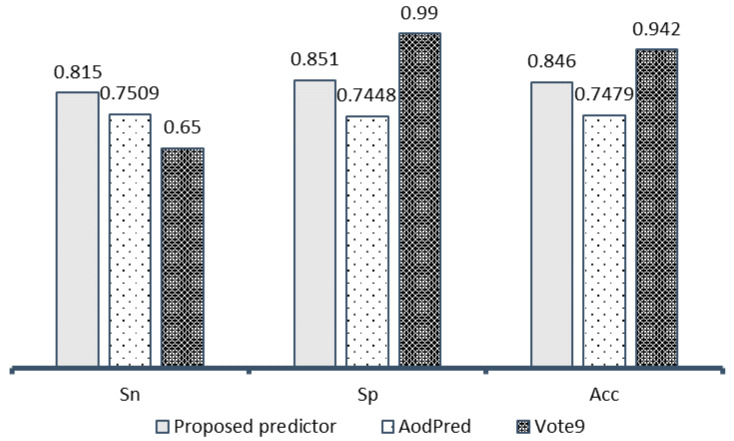
Comparison with existing models on cross-validation.

To further evaluate the predictive performance of the proposed predictor, we compared our model with other existing models, specifically, AodPred [16] and Vote9 [18]. We choose these predictors because they used a benchmarking dataset similar to us, so it should be possible to make a fair comparison in Figure 4.

It is found that our model achieves higher results than AodPred in all metrics: Sensitivity (Sn), Specificity (Sp), and Accuracy (Acc). In the comparison with predictor Vote9, the accuracy of the proposed model is lower, however, the sensitivity in our model is better and balances with the specificity.

#### 3.3.2. Comparison with Existing Models in the Independent Dataset 1

The independent dataset 1 was collected from the research of Butt et al. [5]. It contained 120 positive antioxidant protein sequences and none negative sequence. We used the same benchmarking dataset with the authors. The results of the test (Table 2) showed that our accuracy using the data as balanced with the “The Cost-Sensitive Classifier” tools was higher (0.9667) than the accuracy achieved without adjusting imbalance (0.5167) and when compared with the original accuracy of Butt et al. (0.4916).

#### 3.3.3. Comparison with Existing Models in the Independent Dataset 2

Independent dataset 2 was the same dataset as Zhang et al. [19]. It contains 74 antioxidant protein sequences and 392 non-antioxidant protein sequences. The results of the predictive model on this data set (Table 3) show a significant difference between correcting the data balance and as opposed to using the independent dataset 1. There was a big difference between the value of sensitivity and specificity when balancing the data (0.986 and 0.469), while the accuracy value just reached 0.5515

### 3.4. Replicating the Results Using Weka Toolkit

In bioinformatics, a novel method should be freely and immediately available to the other researchers. Therefore, we used this section to describe how people could replicate our methods using the Weka toolkit. We provide the datasets and optimal models in the supplementary file, and the instructions for replicating are shown as follows:

(1) Extracting features via iFeature toolkit [21].
(2) Selecting the best feature sets that have been provided in Figure 2.
(3) Using Weka to load our provided best model. To prevent any incompatible error, it is suggested to use Weka version 3.8.
(4) Testing your protein sequences based on the generated features in step 2.

Finally, the users could see the results on the submitted sequences that belong to antioxidant proteins or not from Weka output.

### 3.5. Validation with Novel Antioxidant Protein Sequences

To check the model validity on new sequences, we applied our predictor on the validation dataset. This set contained 2934 newly discovered antioxidant proteins that have been described in the dataset section. These proteins were manually annotated via “keyword” search from UniProt/TrEMBL and were related to the antioxidant process. Encouragingly, our predictor achieved an accuracy of 95.06% on this validation dataset. This result, therefore, indicates that our proposed method can be a useful tool for detecting novel antioxidant proteins. In the future, it can be used to validate any unreviewed antioxidant protein as well as accurately predict the antioxidant proteins from unknown sequences.

## 4. Discussion

In this research paper, we found out that Weka [29] is an extremely useful tool in applying both machine learning and deep learning models in the creation of antioxidant protein predictors. First, selecting features by using tree-derived feature importance is a very straightforward, fast, and generally accurate way of selecting good features for machine learning cause reducing the impurity features will help to enhance the calculation ability on the remaining features. In this study, we used the Random Forests method to recursively select the best nine feature sets based upon accuracy (≥0.7) from those seventeen initially considered for inclusion in our models.

For machine learning methods, the main difficulty is when training models with large amounts of data such as protein/peptide sequences resulting in overload. Therefore, in this study, we performed hybridization with attributes of the nine selected feature sets to enhance the accuracy of models and remove the irrelevant factors (reducing the accuracy). When applying all the models to all three-feature datasets, we could see that using the “select attributes” function on all the data features after hybridization (F2) was more efficient than not applying the function (F1), and less efficient than applying the function to each feature set (F3). In general, applying Weka’s filtering function to each feature set and then hybridized will yield high and stable results in both machines learning and deep learning models

In the comparison with previous studies, we validated our optimal model with two independent datasets. For independent dataset 1, the imbalance data adjustment improved the accuracy. Contrary to dataset 1, the adjustment on independent dataset 2 reduced the accuracy of the prediction but increased sensitivity. We believe that the reason comes from the difference between positive and negative data in our training dataset. This does not diminish the value of our prediction model, because the sensitivity is more important than the specificity in the detection of antioxidant protein sequences as our research purpose.

Despite the same dataset was used with other authors, the novelty in our research is imbalance data calibration, which was not performed in the research of Butt et al. [5] as well as Zhang et al. [19], whose research we compared our data with. We also tried building our model in the same methodology with them, without adjustment of imbalance data, for equality in comparison. Our predictor model is still better than the aforementioned authors when performing the testing dataset: accuracy 51.67% vs. 49.16% by Butt et al. [5]; 94.21% vs. 86.3% by Zhang et al. [19], along with 94.6% sensitivity and 94.1% specificity. These results show the significant advantages of our predictor.

## 5. Conclusions

In this study, we have developed an effective model for predicting antioxidant proteins using a series of algorithms that are integrated into the Weka software. We have conducted relevant and irrelevant features screening through the Random Forest classifier. At the same time, we also selected attributes and hybridized features to improve predictive results in machine learning and deep learning models. Finally, among applying different methods, we have achieved the optimal model with 84.6% accuracy, 81.5% sensitivity, and 85.1% specificity for antioxidant protein classifications on the training dataset. Compared to previous studies on the same dataset, our performance was superior to the others.

## Figures and Tables

**Table 1:** Properties of feature sets (descriptors) were used in this research.

Feature Extraction Algorithm	Feature Set (Descriptor)	Dimensions (Amounts of Attributes)
Amino acid composition	AAC: Amino acid composition	20
	CKSAAP: Composition of k-spaced amino acid pairs	2400
	DPC: Dipeptide composition	400
	DDE: Dipeptide deviation from expected mean	400
C/T/D	CTDC: C/T/D Composition	39
	CTDD: C/T/D Distribution	195
	CTDT: C/T/D Transition	39
Conjoint triad	CTriad: Conjoint triad	343
Autocorrelation	Geary: Geary	240
	Moran: Moran	240
	NMBroto: Normalized Moreau-Broto	240
Pseudo-amino acid composition	PAAC: Pseudo-amino acid composition	50
	APAAC: Amphiphilic Pseudo-amino acid composition	80
Grouped amino acid composition	CKSAAGP: Composition of k-spaced amino acid group pairs	150
	GAAC: Grouped amino acid composition	5
	GDPC: Grouped dipeptide composition	25
Quasi-sequence-order	QSOrder: Quasi-sequence-order descriptors	100

**Table 2:** Comparison results on the independent dataset 1.

Predictor	Accuracy
With adjusting imbalance data	$\frac{116}{120}=0.9667$
Without adjusting imbalance data	$\frac{62}{120}=0.5167$
Butt et al. [5] (without adjusting imbalance data)	$\frac{59}{120}=0.4916$

**Table 3:** Comparison results on the independent dataset 2.

Predictor	Sn	Sp	Acc	MCC	AUC
With adjusting imbalance data	0.986	0.469	0.5515	0.341	0.983
Without adjusting imbalance data	0.946	0.941	0.9421	0.811	0.981
Zhang et al., (2016) (without adjusting imbalance data)	0.878	0.860	0.863	0.617	0.948

## Supplementary Materials

The following are available online at https://www.mdpi.com/2079-7737/9/10/325/s1, The following are available online: “data.xlsx” contains our training and independent datasets in FASTA format, “final_model.model” is the optimal model that can be opened in Weka (version 3.8).
